# Silicone-Induced Granulomas of Breast Implant Capsule (SIGBIC)

**DOI:** 10.5334/jbsr.3158

**Published:** 2023-06-08

**Authors:** Evelien Martens, Machteld Keupers

**Affiliations:** 1 University Hospitals Leuven, BE

**Keywords:** breast implants, silicones, granuloma

## Abstract

**Teaching point:** A hypoechogenic mass within the fibrous capsule of a breast implant could correspond with a silicone-induced granuloma.

## Case History

**Figure 1:**
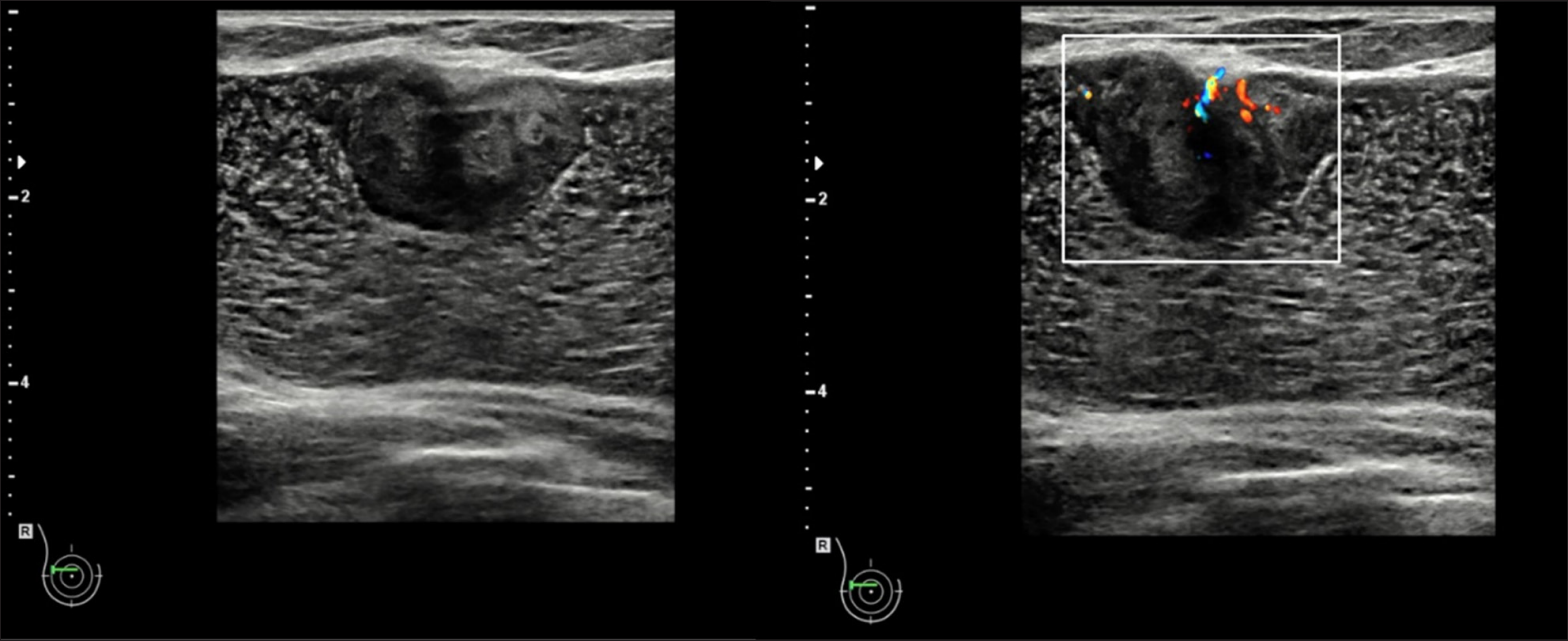


A 61-year-old asymptomatic woman presented at the mammography unit for a routine breast cancer screening. She underwent a breast augmentation with silicone breast implants and free silicone breast injections in 1997. Mammography showed prepectoral breast implants and bilateral several extremely dense lobulated masses with rim calcification, consistent with free silicone deposits. On ultrasound, a circumscribed hypoechogenic mass of 2.5 cm with internal vascularisation was observed between the fibrous capsule and breast implant in the upper outer quadrant (UOQ) of the right breast (Figure 1). The mass increased in volume compared to the previous ultrasound study. Breast magnetic resonance imaging (BMRI) was recommended to differentiate the mass.

**Figure 2:**
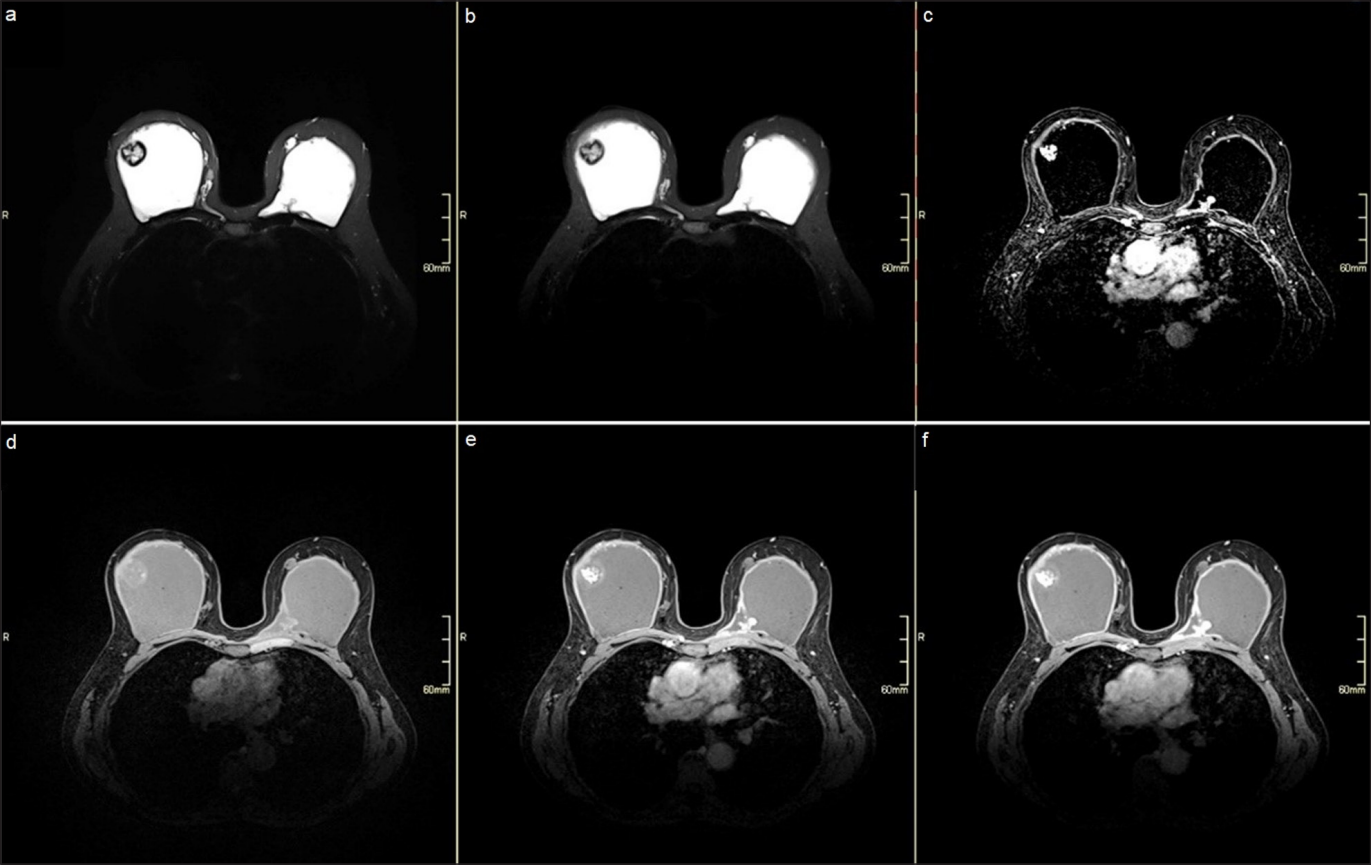


BMRI showed a mass with a heterogeneous T2 hyperintense centre and T2 hypointense peripheral rim extending from the fibrous capsule and protruding in the implant on the T2-weighted and silicone-only sequences (Figure 2a–b). The mass showed central early and continuous contrast enhancement on the dynamic contrast enhanced sequences (Figure 2d–f) and a peripheral fibrous ring without enhancement on the subtraction images (Figure 2c). The extracapsular silicone granulomas caused by the free silicone injections show no contrast enhancement. Bilateral thickening and contrast enhancement of the fibrous capsule was detected, primarily posterior to the implant in the upper inner quadrant (UIQ) of the left breast. Because the mass in the UOQ of the right breast increased in volume and showed central contrast enhancement on BMRI, a biopsy was performed to exclude breast implant-associated anaplastic large cell lymphoma (BIA-ALCL). Histology confirmed the diagnosis of a silicone-induced granuloma. The multidisciplinary board advised surgically removing of the breast implants and a capsulotomy, but the patient preferred follow-up.

## Comments

Breast implant-associated complications have been increasingly reported, including BIA-ALCL and silicone-induced granulomas of breast implant capsule (SIGBIC). SIGBIC typically presents as a mass within the capsule surrounding the breast implant. It is triggered by silicone bleeding, which causes the accumulation of silicone particles in the capsule and a granulomatous inflammation as part of a chronic inflammatory foreign body-like reaction.

On BMRI, SIGBIC can present as a well-defined, heterogeneous T2 hyperintense mass within the fibrous capsule of the breast implant and external compression on the implant. On the dynamic imaging sequence, a black-drop sign can be present as a marked low signal focus at the interface between the mass and the implant, which corresponds to silicone granules within the implant fibrous capsule. They typically show progressive contrast enhancement, and early contrast enhancement of the mass can be seen in extracapsular extension [1]. SIGBIC can be associated with seroma, which can complicate the differential diagnosis with BIA-ALCL as this typically presents with fluid accumulation surrounding the implant. Puncture is advised to differentiate the two entities.
